# IL-1β augments TGF-β inducing epithelial-mesenchymal transition of epithelial cells and associates with poor pulmonary function improvement in neutrophilic asthmatics

**DOI:** 10.1186/s12931-021-01808-7

**Published:** 2021-08-03

**Authors:** Shengding Zhang, Yu Fan, Lu Qin, Xiaoyu Fang, Cong Zhang, Junqing Yue, Wenxue Bai, Gang Wang, Zhihong Chen, Harld Renz, Chrysanthi Skevaki, Xiansheng Liu, Min Xie

**Affiliations:** 1 Department of Respiratory and Critical Care Medicine, Shanxi Bethune Hospital, Shanxi Academy of Medical Sciences, Tongji Shanxi Hospital, Third Hospital of Shanxi Medical University, Taiyuan, 030032 China; 2 Department of Respiratory and Critical Care Medicine, Tongji Hospital, Tongji Medical College, Huazhong University of Science and Technology, Wuhan, China; 3 Key Laboratory of Respiratory Diseases, National Ministry of Health of the People’s Republic of China and National Clinical Research Center for Respiratory Disease, Wuhan, China; 4 Department of Respiratory and Critical Care Medicine, Qiandongnanzhou People’s Hospital, Kaili, China; 5 Department of Respiratory and Critical Care Medicine, Clinical Research Center for Respiratory Disease, West China Hospital, Sichuan University, Chengdu, China; 6 Department of Respiratory and Critical Care Medicine, Zhongshan Hospital, Fudan University, Shanghai, China; 7 Institute of Laboratory Medicine, Philipps Universität Marburg, Marburg, Germany; 8 Universities of Giessen and Marburg Lung Center (UGMLC), and the German Center for Lung Research (DZL), Marburg, Germany

**Keywords:** Asthma, Epithelial-mesenchymal transition, IL-1β, Pulmonary function, TGF-β1

## Abstract

**Background:**

Neutrophilic asthmatics (NA) have less response to inhaled corticosteroids. We aimed to find out the predictor of treatment response in NA.

**Methods:**

Asthmatics (n = 115) and healthy controls (n = 28) underwent clinical assessment during 6-month follow-up with standardized therapy. Asthmatics were categorized by sputum differential cell count. The mRNA expressions were measured by RT-qPCR for sputum cytokines (IFN-γ, IL-1β, IL-27, FOXP3, IL-17A, and IL-5). The protein of IL-1β in sputum supernatant was detected by ELISA. Reticular basement membranes (RBM) were measured in the biopsy samples. The role and signaling pathways of IL-1β mediating the epithelial-mesenchymal transition (EMT) process were explored through A549 cells.

**Results:**

NA had increased baseline sputum cell IL-1β expression compared to eosinophilic asthmatics (EA). After follow-up, NA had less improvement in FEV_1_ compared to EA. For all asthmatics, sputum IL-1β mRNA was positively correlated with protein expression. Sputum IL-1β mRNA and protein levels were negatively correlated to FEV_1_ improvement. After subgrouping, the correlation between IL-1β mRNA and FEV_1_ improvement was significant in NA but not in EA. Thickness of RBM in asthmatics was greater than that of healthy controls and positively correlated with neutrophil percentage in bronchoalveolar lavage fluid. In vitro experiments, the process of IL-1β augmenting TGF-β1-induced EMT cannot be abrogated by glucocorticoid or montelukast sodium, but can be reversed by MAPK inhibitors.

**Conclusions:**

IL-1β level in baseline sputum predicts the poor lung function improvement in NA. The potential mechanism may be related to IL-1β augmenting TGF-β1-induced steroid-resistant EMT through MAPK signaling pathways.

*Trial registration:* This study was approved by the Ethics Committee of Tongji Hospital, Tongji Medical College, Huazhong University of Science and Technology (IRB ID: 20150406).

**Supplementary Information:**

The online version contains supplementary material available at 10.1186/s12931-021-01808-7.

## Background

Asthma is a heterogeneous respiratory disease involving with airway inflammation and hyperresponsiveness. Although eosinophil cell count could be a very good marker to predict therapy response in type-2 asthma, it is not clear which innate biomarkers could predict the poor improvement in non-type-2 asthma patients. Interleukin (IL)-1β is a typical innate immune cytokine which can be mediated by inflammasomes [1]. A recent study shows both leucine-rich repeat-containing family protein 3 inflammasome activation and IL-1β gene expression increase in neutrophilic asthma [2].

Respiratory symptoms and lung function tests are very important for asthma patient evaluation. According to GINA2020, lung function should be evaluated regularly for asthma patients [3]. Low forced expiratory volume in one second (FEV_1_) is a strong independent predictor for acute exacerbations [4, 5] and lung function decline [6]. Since the correlation between lung function and symptoms is weak, it is suggested to monitor lung function in addition to symptoms when evaluating the efficiency of asthma therapy [7].

Airway remodeling has been implicated in persistent airflow obstruction, irreversible decline of lung function, and increased airway hyper-responsiveness in asthma [8]. Airway remodeling is considered the result of repetitive injury caused by chronic airway inflammation. However, the relation between chronic airway inflammation and airway remodeling remains unclear. Epithelial-mesenchymal transition (EMT), a dynamic process by which epithelial cells lose their original epithelial phenotype and transformed into cells with a mesenchymal phenotype, has been demonstrated to play an essential role in airway remodeling. Previous studies have shown that a variety of inflammatory factors, including innate immune cytokine and type 2 cytokines, are involved in EMT [9], that may contribute to airway remodeling and then leads to poor asthma control.

To investigate the values of typical innate immune biomarkers and type-2 biomarkers in predicting the response to asthma therapy and the potential mechanisms, we conducted a prospective study. Our results indicated that IL-1β level in induced sputum of untreated patients predicts the poor lung function improvement in neutrophilic asthmatics. The potential mechanism is related to IL-1β augmenting TGF-β1 induced EMT.

## Methods

### Subjects and study design

Adults patients with physician diagnosed asthma, according to the Global Initiative for Asthma 2014, were recruited if they met the following key criteria: bronchoprovocation test with methacholine PD20 < 2.505 mg or bronchodilation FEV_1_ change > 200 ml and 12%; not received treatment with any inhaled or systemic glucocorticoids in the previous three months; and successfully induced sputum at baseline during June 15, 2015-April 20, 2017 from the Department of Respiratory and Critical Care Medicine at Tongji Hospital (Wuhan, China) of Huazhong University of Science and Technology. The exclusion criteria included: acute episode in recent one month; respiratory infection in the last two weeks; comorbid with chronic obstructive pulmonary disease; bronchiectasis; other respiratory disease; pregnancy; or serious organ failure. Nonsmokers were defined as never smokers or ex-smokers who had the smoking history of less than five pack-years and quit smoking for longer than six months, otherwise defined as smokers. Atopy was defined as at least one specific IgE (≥ 0.35 kUI/L) toward common aeroallergens, a positive skin prick test response, or both. Healthy controls had no history of chronic respiratory disease; no respiratory infection within two weeks; no history of atopy; no family history of asthma; no smoke; no severe systemic disease.

Participants at the baseline visit (labeled as visit 1, V1) underwent clinical characteristics assessment, spirometry and sputum induction. In the follow-up, only those received 6-month standardized therapy with good adherence were recorded ACT score and pulmonary function test at the end of 6-month treatment (labeled as visit 2, V2). The improvement of FEV_1_ was calculated in three ways [10]: (1) absolute change in FEV_1_, calculated by the difference between FEV_1_ after six months treatment and baseline FEV_1_, labeled as ΔFEV_1(V2-V1)_; (2) FEV_1_ change ratio, calculated by absolute change in FEV_1_ divided by baseline FEV_1_, labeled as ΔFEV_1(V2-V1)_/FEV_1(V1)_ baseline; (3) absolute change in percentage of the predicted FEV_1_, calculated by the difference between percentage of the predicted FEV_1_ after six months treatment and baseline, labeled as ΔFEV_1_%_(V2-V1)_.

All participants were given a written informed consent prior to clinical data and sample collection. The study was approved by the Ethics Committee of Tongji Hospital, Tongji Medical College, Huazhong University of Science and Technology (IRB ID: 20150406).

### Sputum induction

Sputum induction was processed and cytospins were prepared as described before [11]. Using sputum eosinophils and neutrophils count, participants were categorized as eosinophilic (eosinophils ≥ 3% and neutrophils < 61%), neutrophilic (neutrophils ≥ 61% and eosinophils < 3%), mixed-granulocytic (neutrophils ≥ 61% and eosinophils ≥ 3%) or pauci-granulocytic asthma (sputum neutrophils < 61% and sputum eosinophils < 3%) [2]. Sputum samples with enough cells for mRNA isolation and less than 3% epithelial cells were processed for gene expression using quantitative reverse transcription (RT-qPCR), sputum supernatant was measured for IL-1β concentration with enzyme-linked immunosorbent assay (ELISA) as previously described [12].

### RT-qPCR and ELISA

Total RNA was extracted from the cell pellets if they were enough. Reverse transcription was performed using the PrimeScript RT reagent Kit (Takara, Dalian, China). The specific PCR primers were synthesized by Riobio Co. Lit. (Guangzhou, China). RT-qPCR assays were performed using the SYBR PremxiExTag (Takara, Dalian, China) on a 7500 RT-qPCR System (Life Technologies, Carlsbad, CA). The relative expression levels were normalized to β-actin and calculated using the 2^−ΔCT^ method.

Sputum supernatant was store in at − 80 °C for subsequent detection. IL-1β in sputum supernatant was measured by Human IL-1beta DuoSet ELISA Kit (R&D Systems Inc. MN, USA) according to the manufacturer’s instruction. The detection limits were 3.91 pg/mL. All values below the detection limits were set as 3.91 pg/mL.

### Bronchoscopy and endobronchial biopsy

Bronchial alveolar lavage cells were obtained bronchoscopically from fourth- to fifth-generation airways of the right middle lobe. Endobronchial biopsy was taken from R8-R9 ridge of the right lower lobe subsegments and fixed in 4% formalin for further hematoxylin–eosin staining, as previously described [13].

### Measurement of the thickness of reticular basement membrane

The section of each endobronchial biopsy was taken 2–5 micrographs for the best preserved mucous membrane and reticular basement membrane (RBM) under microscope with Qian Ping image system. The thickness of RBM was calculated by dividing the area by the length of RBM using an image analysis system (Image Plus Pro), as previously described [13].

### Cell culture and cytokine stimulation

The A549 cells were grown in DMEM (HyClone, CT, USA) supplemented with 10% fetal bovine serum (Gibco, Australia). The cells would be put in DMEM medium with 1% low concentration fetal bovine serum for 24 h before the cytokines stimulation. 10 ng/ml IL-4, 5 ng/ml transforming growth factor (TGF)-β1, and 10 ng/ml IL-1β (PeproTech, Princeton, USA) were used separately or in combination to stimulate the cells. The stimulation would last for 24 h to observe morphological change of A549 cells or detect mRNA expression of EMT markers, or 48 h to detect protein expression EMT markers, or for 1 h to detect the phosphate kinase expression. Before the cytokines stimulation, cells were pretreated for 1 h with MEK inhibitor U0126, p-JNK1/2 inhibitor SP600125, or p38 inhibitor SB203580 (10 μM, MCE, Shanghai, China) if necessary. To detect the effect of glucocorticoid and montelukast sodium on TGF-β1 combined with IL-1β-induced EMT in A549 cells, methylprednisolone (Sigma, USA) or montelukast sodium (Merck, NJ, USA) were dissolved in phosphate buffer saline, then cells were pretreated for 24 h with methylprednisolone (0.5 mg/ml) or montelukast sodium (0.01 μM) followed by TGF-β1 combine with IL-1β stimulation.

### Western blot

RIPA lysis buffer (Aspenbio, Wuhan, China) was used to extract total cellular protein. The primary antibodies including E-cadherin, Fibronectin (1:2000 dilution, Proteintech, Wuhan, China), p-ERK1/2, p-JNK, p-p38, p-AKT, p-NF kB, and p-smad3 (1:1000 dilution, Cell Signaling Technology, Danvers, MA, USA) were incubated in 4 °C overnight. Goat anti-rabbit IgG and Goat anti-mouse IgG (1:4000 dilution, Aspenbio, Wuhan, China) were used as secondary antibodies. The protein expression levels were normalized to GAPDH or β-actin (1:2000, Sungenebiotech, Tianjin, China).

### Statistical analyses

Data were expressed as means ± standard deviation or medians with interquartile range. Sputum gene expression levels were log-transformed. Parametric continuous variables were tested by Student’s t-test. Multiple groups were compared using one-way analysis of variance with a Bonferroni correction (normal data) or a Kruskal–Wallis test with a Dunn intergroup comparison (non-normal data). Categorical variables were analyzed using Chi-Square or Fisher exact test. Spearman’s rank correlation coefficient was used for correlation analyses. *P* < 0.05 was considered statistically significant. SPSS software V.20.0 was used for analyses.

## Results

### Subjects characteristics

Baseline characteristics of the subjects were summarized in Table 1. 28 healthy controls and 115 asthmatics were enrolled with induced sputum samples. Identified by sputum differential cell counts, asthmatic patients were subgrouped into eosinophilic (n = 32), neutrophilic (n = 30), mixed (n = 11) and pauci-granulocytic (n = 42) asthmatics. Although healthy controls were younger than asthmatic patients, the differences of age, sex, body mass index (BMI), smoking status, and baseline lung function among the four asthmatic subgroups were not significant. The eosinophilic group was presented with higher levels of blood eosinophil counts, higher exhaled NO and higher levels of total IgE than the neutrophilic group at the baseline visit. More details were also summarized in Table 1.

**Table 1:** Baseline demographic characteristics of all groups (n = 143)

		Asthmatic patients				
	Healthy (n = 28)	Eosinophilic (n = 32)	Neutrophilic (n = 30)	Mixed (n = 11)	Paucigranulocytic (n = 42)	Overall *P* value
Age (y)	25 (23–26)	44 (27–50)^§^	43 (34–51)^§^	49 (41–51)^§^	46 (31–52)^§^	< 0.001
Male sex n. (%)	14 (50)	20 (63)	9 (30)	8 (73)	17 (41)	0.035
BMI (kg/m^2^)	20.3 (18.9–23.5)	22.5 (20.7–24.6)	22.3 (20.3–23.4)	23.7 (22.3–27.2)	22.4 (18.7–25.6)	0.152
Smoker n. (%)	0 (0)	10 (31)^§^	5 (17)^§^	3 (27)^§^	13 (31)^§^	0.016
Atopy n. (%)	0 (0)	18 (56)^§^†	9 (30)^§^	4 (36)^§^	16 (38)^§^	< 0.001
Asthma course (y)	NA	2.0 (1.0–7.5)	2.8 (1.0–8.0)	4.0 (2.0–11.0)	1.0 (0.25–7.0)	0.121
Blood eosinophils (%)	1.8 (1.3–2.7)	6.4 (3.3–8.8)^§†^£	2.6 (0.9–4.6)	5.5 (2.4–7.9)^§^£	2.1 (1.2–3.2)	< 0.001
Blood neutrophils (%)	56.7 (53.7–61.5)	54.6 (49.2–61.7)	61.2 (53.6–65.6)	59.7 (55.6–66.1)	56.3 (52.7–63.3)	0.356
FEV_1_ (L)	3.35 ± 0.55	2.55 ± 0.64^§^	2.59 ± 0.79^§^	2.66 ± 0.86	2.64 ± 0.59§	< 0.001
FEV_1_ (%)	90.9 ± 7.2	82.8 ± 17.1	90.1 ± 18.2	83.7 ± 19.3	90.1 ± 13.2	0.163
FEV_1_/FVC (%)	87.3 ± 6.1	67.0 ± 8.9§	71.9 ± 11.8§	64.3 ± 9.7§	73.2 ± 10.1§	< 0.001
Serum IgE (IU/ml)	40 (12–66)	215 (60–519)^§£^	92 (36–254)	118 (78–410)	52 (14–193)	< 0.001
FE_NO_ (ppb)	NA	86 (30–112)^†£^	27 (17–45)	48 (31–85) ^£^	23 (11–33)	< 0.001
ACT score	NA	16.0 (14–18)^£^	16.5 (13–19)	16.0 (14–19)	18.0 (16–19)	0.038
Induced sputum characteristics^‡^						
Macrophages (%)	37.3 (28.1–50.2)	22.1 (11.5–35.8)^£^	8.9 (4.3–18.2)^§^k^£^	4.6 (3.4–9.4)^§k£^	47.7 (37.1–58.4)	< 0.001
Neutrophils (%)	51.3 (43.2–64.7)	31.7 (18.3–45.7)^§†¶^	81.7 (68.6–89.6)§^£^	74.1 (64.6–84.9) ^£^	39.1 (30.3–46.0)	< 0.001
Eosinophils (%)	0.1 (0–0.2)	21.1 (7.7–43.1)^§†£^	0.5 (0.1–1.6)	8.1 (5.0–15.1)^§†£^	0.2 (0–0.9)	< 0.001
Lymphocytes (%)	4.1 (2.6–6.8)^k£^	8.1 (5.0–13.7)	4.7 (3.1–10.7)	4.6 (2.7–10.1)	7.8 (5.5–13.8)	0.002

Normal data are expressed as mean ± SD and non-normal data are described as median (IQR). Multiple groups were compared using one-way analysis of variance (ANOVA) with a Bonferroni correction (normal data) or a Kruskal–Wallis test with a Dunn intergroup comparison (non-normal data). The Levene method was used to test for multiple-sample homogeneity of variance, and Welch method was performed when data are heterogeneous. The χ^2^ or Fisher exact tests were used to compare ratios;

*BMI* body mass index, *FE*_*NO*_ fraction of exhaled nitric oxide, *ACT* Asthma Control Test, *IQR* interquartile range, *NA* not available

Atopy was defined aswas defined as at least one specific IgE (≥ 0.35 kUI/L) toward common aeroallergens, a positive skin prick test response, or both

Smoker was defined as current smokers or ex-smokers who had the smoking history of more than 5 pack-years or quit smoking for less than 6 months

^‡^Data were missing for five patients in healthy group

^§^*p* < 0.05 versus healthy subjects

^k^*p* < 0.05 versus patients with eosinophilic asthma

^†^*p* < 0.05 versus patients with neutrophilic asthma

^¶^*p* < 0.05 versus patients with mixed asthma

^£^*p* < 0.05 versus patients with Paucigranulocytic asthma

### Sputum profile of asthmatic patients

**Fig. 1:**
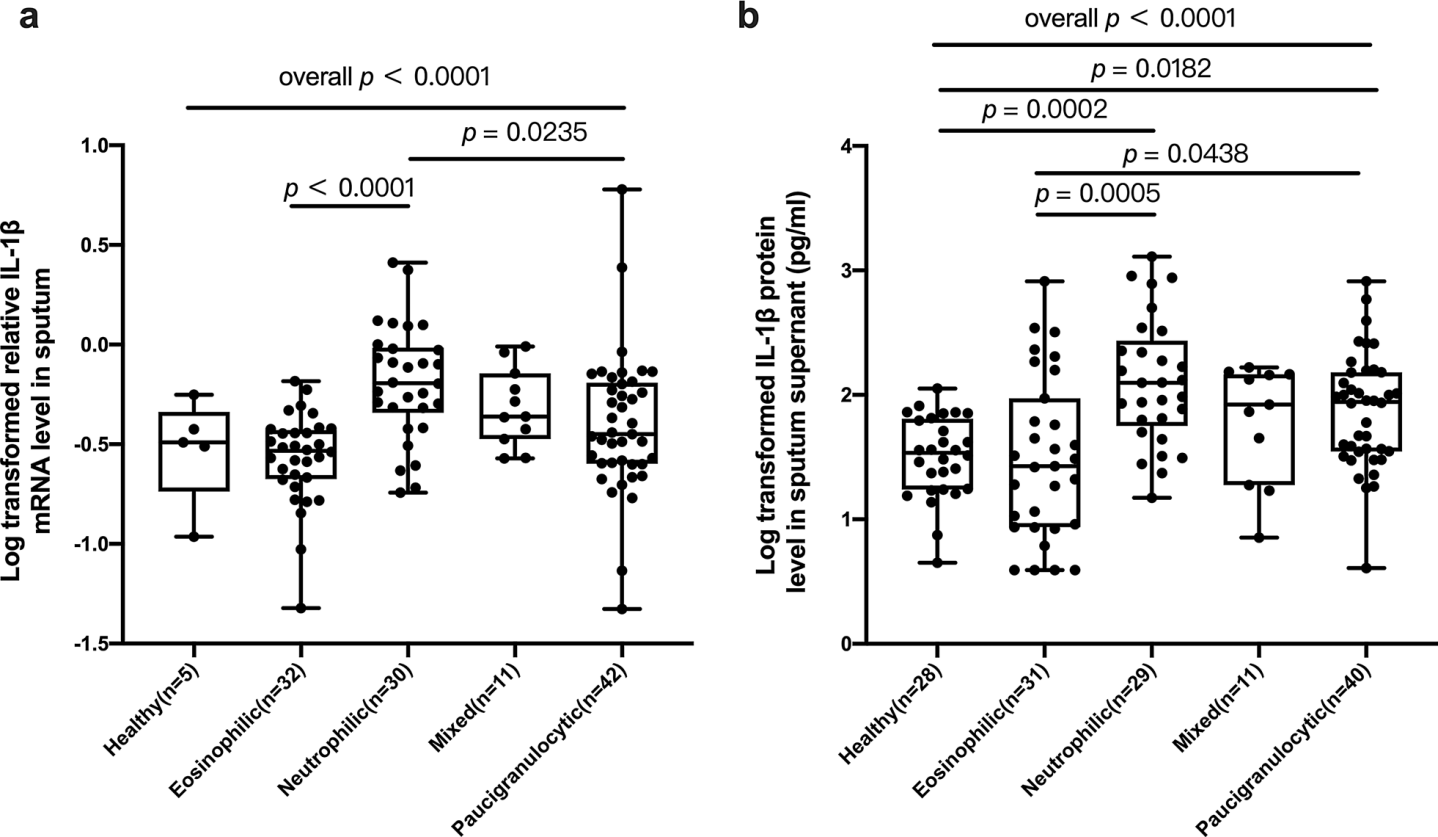
The baseline **a** sputum cell IL-1β mRNA levels and **b** IL-1β protein levels in sputum supernatant among healthy controls and four subgroups of asthmatic patients. The data are presented as dot plots, mRNA and protein expression were log transformed

The eosinophilic group had higher baseline sputum cell IL-5 mRNA expression compared to neutrophilic (*p* = 0.004) and pauci-granulocytic groups (*p* < 0.001) (Additional file 1: Figure S1b). Mixed-granulocytic group had higher baseline sputum cell IL-17A mRNA expression compared to neutrophilic (*p* = 0.005) and pauci-granulocytic groups (*p* = 0.020) (Additional file 1: Figure S1d). Neutrophilic group had higher baseline sputum cell IL-1β mRNA expression compared to eosinophilic (*p* < 0.0001) and pauci-granulocytic groups (*p* = 0.024) (Fig. 1a). Neutrophilic group had higher levels of IL-1β protein in sputum supernatant than healthy controls (*p* < 0.001) and eosinophilic groups (*p* < 0.001). There was no significant difference of the baseline mRNA level of sputum IFN-γ, IL-27 or FOXP3 mRNA expression among the four subgroups (Additional file 1: Figure S1a, b, and e).

As is shown in Additional file 2: Table S1, IL-1β mRNA expression was positively correlated with neutrophil percentage (R = 0.459, *p* < 0.0001), but negatively correlated with eosinophil percentage (R = − 0.224, *p* = 0.017) and macrophage percentage (R = − 0.230, *p* = 0.014). IL-27 mRNA expression was positively correlated with IFN-γ (R = 0.658, *p* < 0.0001) and IL-5 mRNA expression (R = 0.442, *p* < 0.0001). IFN-γ mRNA expression was positively correlated with IL-5 mRNA expression (R = 0.564, *p* < 0.0001). Eosinophil percentage was positively correlated with IL-5 mRNA expression (R = 0.370, *p* < 0.0001), but negatively correlated with neutrophil percentage (R = − 0.196, *p* = 0.035) and macrophage percentage (R = − 0.270, *p* = 0.003).

### Poor pulmonary function improvement in neutrophil asthmatic patients

54 patients were followed up for six months with standardized asthma therapy. Baseline demographic characteristics of these patients are summarized in Additional file 3: Table S2. There was no significant difference in age, sex, BMI, smoking status, or baseline lung function among the four asthmatic groups.

**Fig. 2:**
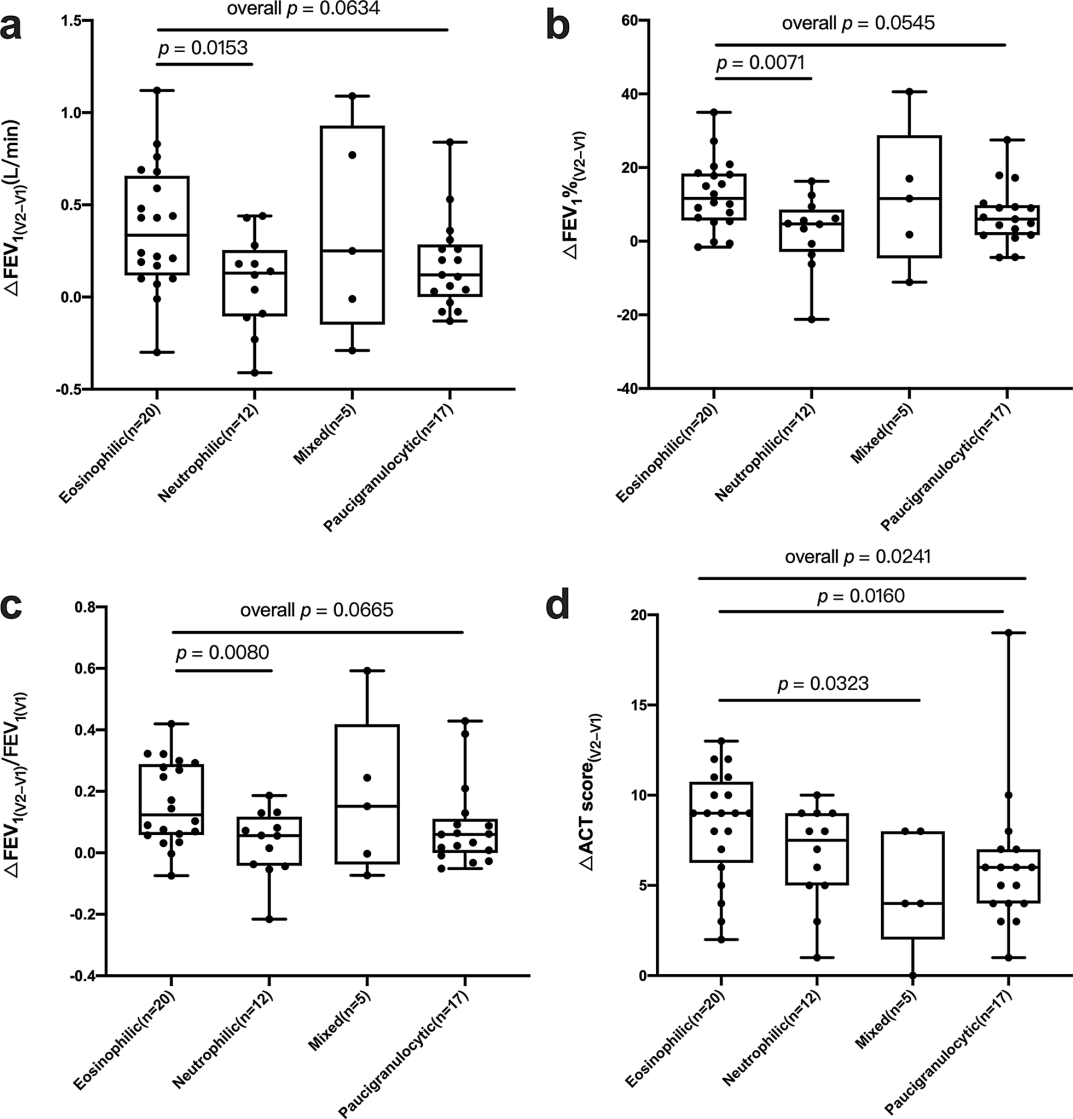
The improvement of lung function **a**–**c** and ACT scores **d** after six months standardized therapy among four subgroups of asthmatic patients. ΔFEV_1(V2-V1)_: absolute change in FEV_1_, calculated by the difference between FEV1 after six months treatment and baseline FEV_1_; ΔFEV_1_%_(V2-V1)_: absolute change in percentage of the predicted FEV_1_, calculated by the difference between percentage of the predicted FEV_1_ after six months treatment and baseline percentage of the predicted FEV_1_; ΔFEV_1(V2-V1)_/FEV_1(V1)_: FEV_1_ change ratio, calculated by absolute change in FEV_1_ divided by baseline FEV_1_. ΔACT_(V2-V1)_, absolute change in ACT, calculated by the difference between ACT after six months treatment and baseline ACT

The neutrophilic asthmatics had less improvement in FEV_1_ as compared with eosinophilic asthmatics (Fig. 2a-c). Mixed-granulocytic and pauci-granulocytic asthmatics had less improvement in ACT scores as compared with eosinophilic asthmatics (Fig. 2d). There was no significant difference in post-treatment FEV_1_ levels or ACT scores among four asthmatic groups (Additional file 4: Figure S2).

**Fig. 3:**
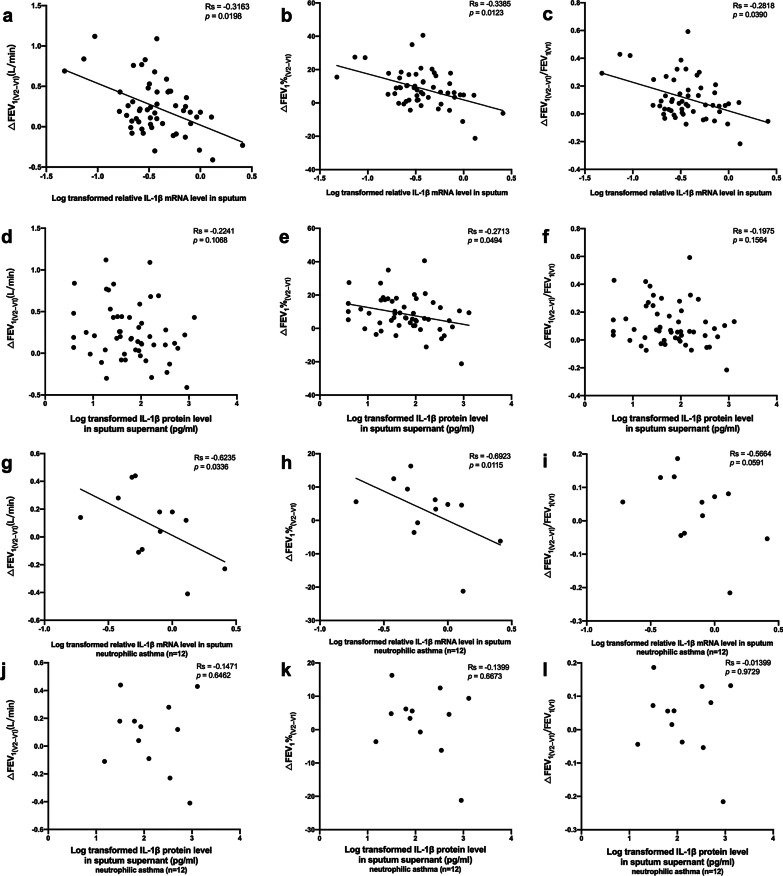
**a**–**c** The correlations of baseline IL-1β mRNA expression with spirometry change in all asthmatic patients. **d**–**f** The correlations of baseline IL-1β protein level in sputum supernatant with spirometry change in all asthmatic patients. **g**–**i** The correlations of baseline IL-1β mRNA expression with spirometry change in neutrophilic asthmatic patients. **j**–**l** The correlations of baseline IL-1β protein level in sputum supernatant with spirometry change in neutrophilic asthmatic patients. The data are presented as dot plots, with fitted regression lines. mRNA and protein expression were log transformed, Spearman R-values and p-values are indicated

For all asthmatic patients (n = 54), baseline sputum IL-1β mRNA was negatively correlated with absolute change in FEV_1_ (R = − 0.316, *p* = 0.020), absolute change in percentage of the predicted FEV_1_ (R = − 0.339, *p* = 0.012), and FEV_1_ change ratio (R = − 0.282, *p* = 0.039) (Fig. 3a–c). Baseline sputum IL-1β mRNA was positively correlated with baseline sputum IL-1β protein expression (R = 0.363, *p* = 0.008). Baseline sputum IL-1β protein expression was negatively correlated with absolute change in percentage of the predicted FEV_1_ (R = − 0.271, *p* = 0.049) (Fig. 3e), but had no significant correlation with absolute change in FEV_1_ and FEV_1_ change ratio (Fig. 3d and f). Eosinophil percentage was positively correlated with absolute change in FEV_1_ (R = 0.383, *p* = 0.004), absolute change in percentage of the predicted FEV_1_ (R = 0.382, *p* = 0.004), and FEV_1_ change ratio (R = 0.387, *p* = 0.004) (Additional file 5: Table S3.1).

Subgroup analysis showed that in neutrophilic asthmatics (n = 12), baseline sputum cell IL-1β mRNA expression was negatively correlated with absolute change in FEV_1_ (R = − 0.624, *p* = 0.034) and absolute change in percentage of the predicted FEV_1_ (R = − 0.692, *p* = 0.012), but had no significant correlation with FEV_1_ change ratio (Fig. 3g–i). IL-1β protein expression had no significant correlation with change in FEV_1_ (Fig. 3j–l). For the eosinophilic asthmatics (n = 20), neither IL-1β mRNA expression nor IL-1β protein expression had significant correlation with change in FEV_1_ (Additional file 5: Table S3.2). For pauci-granulocytic asthmatics (n = 17), IL-1β protein expression was negatively correlated with absolute change in percentage of the predicted FEV_1_ (R = − 0.495, *p* = 0.045), but had no significant correlation with absolute change in FEV_1_ and FEV_1_ change ratio (Additional file 5: Table S3.4). More details were also summarized in Additional file 5: Table S3.

### The thickness of reticular basement membrane in asthma correlated with neutrophil percentage in BALF

29 asthmatic patients and four healthy controls underwent bronchoscopy and yield sufficient tissue to evaluate the RBM thickness. The thickness of RBM in asthma patients was significantly greater than that of healthy controls (9.6 ± 3.9 μm vs 4.4 ± 1.1 μm, *p* = 0.003) (Additional file 6: Figure S3a). The thickness of RBM in asthmatics was positively correlated with neutrophil percentage in bronchoalveolar lavage fluid (BALF) (R = 0.414, *p* = 0.032), but had no significant correlation with eosinophil percentage in blood and eosinophil percentage in BALF (Additional file 6: Figure S3b-d).

### IL-1β augmented TGF-β1 inducing epithelial-mesenchymal transition of epithelial cells

**Fig. 4:**
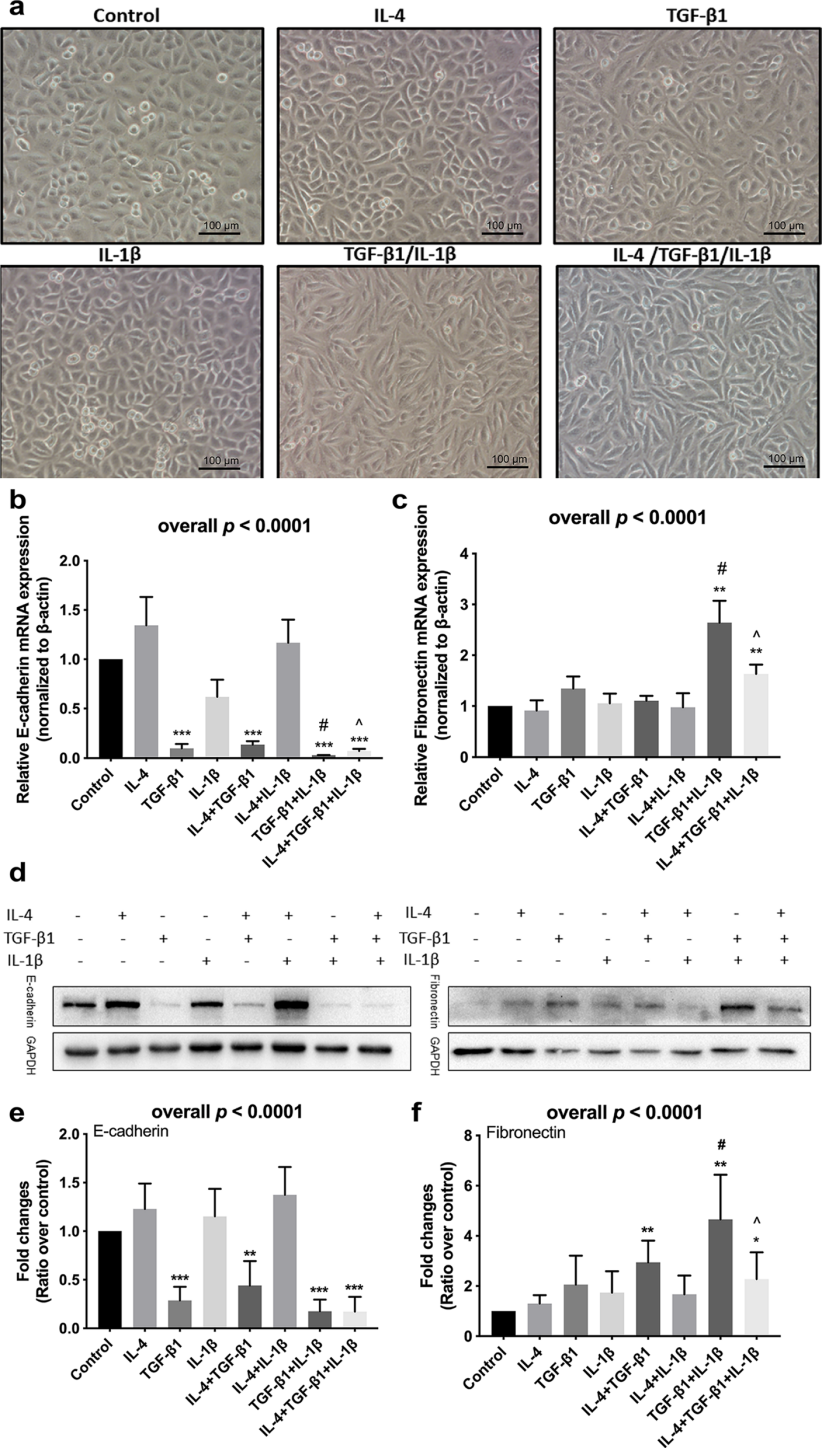
The effect of IL-1β on TGF-β1-induced EMT in asthmatic A549 cells. **a** Morphological change of A549 cells (magnification × 100). The relative **b** E-cadherin and **c** Fibronectin mRNA expression were assessed by means of quantitative real-time PCR. Expression levels were normalized to the housekeeping gene β-actin and calculated over untreated control cells. **d** Western blot analyses of E-cadherin and Fibronectin in A549 cells. The relative **e** E-cadherin and **f** Fibronectin protein expression were assessed by means of western blot. Expression levels were normalized to the housekeeping gene GAPDH and calculated over untreated control cells. The figures **b**, **c** illustrate cumulative data from 3 independent experiments. The figures **e**, **f** illustrate cumulative data from 5 independent experiments. A549 cells were cultured in the absence (−) or presence (+) of IL-4, TGF-β1, and IL-1β. Error bars represent standard deviation. *p < 0.05, **p < 0.01, ***p < 0.001compared with control group. #*p* < 0.05 compared with TGF-β1 treated group.^*p* < 0.05 compared with TGF-β1 combined with IL-1β treated group

Under microscopy observation, a part of A549 cells developed a spindle fibroblast-like morphology after treatment with TGF-β1. While, treatment with IL-1β alone could not induce the morphological change, TGF-β1 in combination with IL-1β would induce a majority of A549 cells transforming into a spindle fibroblast-like morphology (Fig. 4a). The expression of an epithelial marker, E-cadherin significantly decreased by TGF-β1 treatment both in mRNA (Fig. 4b) and protein level (Fig. 4d and e) in A549 cells. The expression of E-cadherin mRNA was significantly lower after stimulated by TGF-β1 in combination with IL-1β compared with treatment with TGF-β1 alone. The decrease of E-cadherin mRNA in A549 cells after stimulation by TGF-β1 in combination with IL-1β could be partially reversed by additional IL-4 (Fig. 4b). The TGF-β1 stimulation induced an increase in Fibronectin mRNA in A549 cells as well as the corresponding changes in the protein levels (Fig. 4). The expression of Fibronectin mRNA was significantly higher after stimulation by TGF-β1 with IL-1β compared with that with TGF-β1 alone. While the expression of Fibronectin was lower after stimulation by IL-4, TGF-β1, and IL-1β compared with that with TGF-β1 and IL-1β (Fig. 4c) as well as the corresponding trends in the protein levels (Fig. 4d and f).

### Neither glucocorticoid or montelukast inhibited EMT induced by IL-1β and TGF-β1 in A549 cells

A549 cells were pretreated with glucocorticoid or montelukast sodium for 24 h and subsequently stimulated with TGF-β1 in combination with IL-1β for two days. While TGF-β1 in combination with IL-1β significantly decreased E-cadherin and increased Fibronectin in A549 cells, neither glucocorticoid or montelukast sodium of pretreatment could inhibit the change of EMT protein expression (Additional file 7: Figure S4).

### MAPK signaling pathways mediated IL-1β augmenting epithelial-mesenchymal transition of epithelial cells

**Fig. 5:**
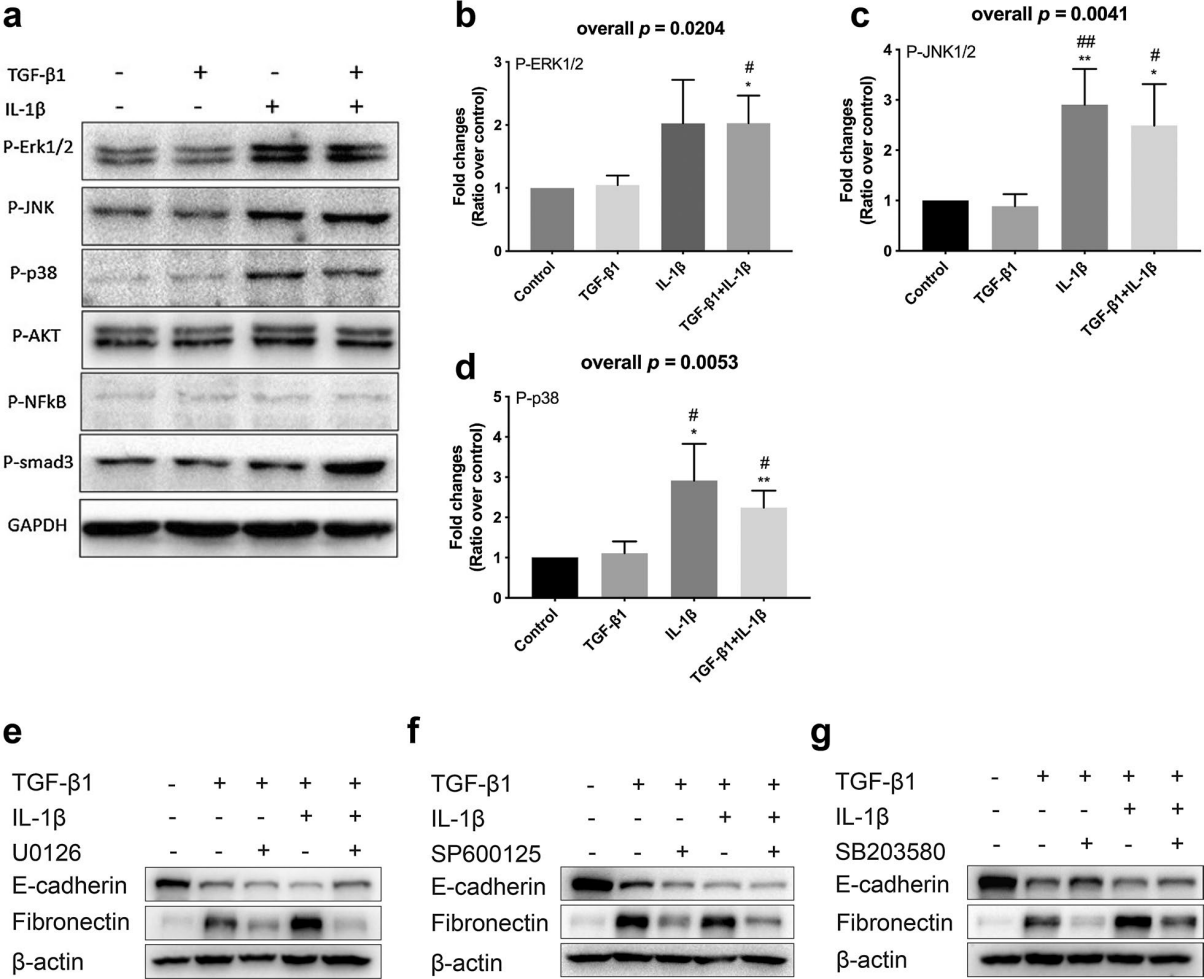
The candidate signaling pathways mediated the process of IL-1β augmenting TGF-β1-induced EMT in A549 cells. A549 cells were cultured in the absence (−) or presence (+) of TGF-β1, IL-1β. **a** Proteomic analyses of p-ERK1/2, p-JNK1/2, p-p38, p-AKT, p-NFκB, and p-smad3. The relative **b** p-ERK1/2, **c** p-JNK1/2, and **d** p-p38 protein expression were assessed by means of western blot. Expression levels were normalized to the housekeeping gene GAPDH and calculated over untreated control cells. Then the inhibitors of **e** MEK, **f** p-JNK1/2, and **g** p-p38 were used to determine if the process of IL-1β augmenting TGF-β1-induced EMT in A549 cells was mediated by MAPK signaling pathways. A549 cells were cultured in the absence (−) or presence (+) of TGF-β1, IL-1β, and three inhibitors of MAPK signaling pathway. The protein expressions of E-cadherin and Fibronectin were assessed by western blot analysis. The figures **b**–**d** illustrate cumulative data from 3 independent experiments. Error bars represent standard deviation. *p < 0.05, **p < 0.01, ***p < 0.001 compared with control group. #*p* < 0.05, ##*p* < 0.01, ###*p* < 0.001 compared with TGF-β1 treated group

To further investigate the mechanism of IL-1β augmenting TGF-β1 inducing EMT, Western Blot was used to detect the expression of candidate signaling pathways related proteins. The results showed that after the stimulation by TGF-β1 with IL-1β, the expression of p-ERK1/2, p-JNK1/2, and p-p38 increased significantly in A549 cells compared with control group and the group stimulated with TGF-β1 alone (Fig. 5a–d). Both MEK inhibitor U0126, p-JNK1/2 inhibitor SP600125, and p38 inhibitor SB203580 significantly inhibited the expression of Fibronectin induced by TGF-β1 in combination with IL-1β (Fig. 5e–g). P38 inhibitor SB203580 could partially reverse the inhibitory effect of TGF-β1 with and without IL-1β on E-cadherin expression in A549 cells (Fig. 5g).

## Discussion

There is increasing evidence that asthma is a heterogeneous inflammatory airway disorder involving Th2-driven and non-Th2-driven mechanisms approximately half by half [14]. Recent studies demonstrate non-Th2 inflammation in asthma may lead to poor control of asthma. Especially, the asthmatic patients who have neutrophil-predominant airway inflammation defined by induced sputum cell counts, had less improvement in lung function and symptoms control after treatment with inhaled corticosteroids [15]. To our best knowledge, this is the first study that revealed higher IL-1β expression in sputum may predict less improvement of lung function in neutrophilic asthma patients.

In this study, we picked cytokines in sputum which represent innate immune response or Th2 immune response. Our findings are consistent with recent studies that the sputum mRNA and protein expression levels of IL-1β were significantly elevated in neutrophilic asthmatics [2] and the sputum mRNA expression levels of IL-5 mRNA were significantly elevated in eosinophilic asthmatic patients which confirmed the effect of IL-5 on eosinophilic inflammation [16]. This consistency shows that our results are reliable. Neutrophilic asthmatics had less improvement in FEV_1_ (calculated in three ways) as compared with eosinophilic asthmatics. The results have shown that baseline sputum IL-1β mRNA and protein expression were negatively correlated with the improvement of lung function, eosinophil percentage was positively correlated with the improvement of lung function for all asthmatics. However, for neutrophilic asthmatics, categorized by sputum eosinophils and neutrophils count, only IL-1β mRNA expression correlated with the less improvement of lung function, indicating that IL-1β may be an important biomarker for poor lung function improvement in neutrophilic asthmatics.

Patients with impaired lung function are inclined to have acute exacerbation, poor quality of life and uncontrolled asthma [17]. However, those neutrophilic asthmatics, which had poor lung function improvement after standardized therapy, had no statistical difference in baseline lung function and symptoms scores compared with asthmatics with other phenotypes, and there is no significant correlation between neutrophil percentage and lung function improvement, means that it is difficult to distinguish asthmatics who may have worse lung function from all patients in the early stage. The results of this study show a possibility that baseline sputum cell IL-1β mRNA expression may be used as a predictive factor for less lung function improvement in neutrophilic asthmatics.

IL-1β elevation has been reported in asthmatics with sputum neutrophilia in several recent studies [2, 18, 19]. Evans et al. have reported that IL-1 receptor as an important predictor of both neutrophilic asthma and worse lung functions, however, IL-1β was not measured in their study [20]. Juan-juan et al. observed a trend of increased IL-1β gene and protein expression in asthmatics with frequent exacerbations [12]. IL-1β was also confirmed to be involved in the viral stimulus-induced asthma exacerbation [21, 22]. Due to the small sample size of asthmatics with at least one exacerbation during six months of follow-up period (only three patients), we could not analyze the relationship between IL-1β mRNA expression and asthma exacerbation in our study. IL-1β and IL-1 signaling were also found to contribute to lung neutrophilic inflammation, which negatively impacts lung function in experimental fungal-associated asthma mice model [23].These studies have shown that IL-1β plays an important role in airway inflammation, especially neutrophil inflammation, in bronchial asthma.

However, it is not clear how IL-1β affects lung function in asthmatic patients. Mehta et al. have found that IL-1β is essential in recurrent rhinovirus infection induced airway remodeling in the absence of allergen [24].This finding suggests that IL-1β may contribute to the decline of lung function by promoting airway remodeling. In our study, we found that mean thickness of RBM in asthma was significantly greater than that of healthy controls. For asthmatic patients, the thickness of RBM was positively correlated with neutrophil percentage in BALF, indicating that airway neutrophilic inflammation may play an important role in airway remodeling. EMT is regarded as an important pathophysiological process in airway remodeling in asthmatic patients [9]. Previous studies have demonstrated that IL-1β can induce EMT or endothelial-to-mesenchymal in a variety of epithelial or endothelial cells, such as hepatocellular carcinoma cells, esophageal squamous cell carcinoma cells, aortic endothelial cells, and so on [25–27]. However, in human bronchial epithelial cell lines the results are inconsistent, a previous study found that stimulation with IL-1β alone cannot induce EMT [28]. Other studies found that IL-1β alone can induce a significant reduction in E-cadherin protein expression [29, 30]. E-cadherin is usually expressed in the cell membrane adhering junctions of epithelial cells, which connects epithelial cells together, the decrease of E-cadherin can be a marker of EMT [31]. In our study, treatment with IL-1β individually could not induce changes in morphological or EMT markers expression in A549 cells. Transforming growth factor-β (TGF-β), a multifunctional cytokine that induces tissue fibrosis, is the main factor responsible for driving EMT [32]. As IL-1β was negatively correlated with the improvement of lung function, we hypothesized that IL-1β augmented TGF-β1 induced EMT of human lung epithelial cells. Our results showed that TGF-β1 induced part of A549 cells developed a spindle fibroblast-like morphology, and IL-1β could significantly enhance this effect. Interestingly, when add together with IL-4, after treatment with IL-4/TGF-β1/IL-1β, the proportion of cell morphological changes seems to have decreased compared with treatment with TGF-β1/IL-1β. At the mRNA and protein level, this effect has been confirmed. These results indicate that IL-4 can partly reverse EMT induced by TGF-β1/IL-1β. This may be one of the reasons why eosinophilic asthmatics had more improvement of lung function after therapy as compared with neutrophilic asthmatics.

Glucocorticoid and leukotriene receptor antagonists (LTRA) are the most commonly used drugs in the therapy of asthma [33].The abilities of dexamethasone and fluticasone propionate to inhibit MMP-2 expression, which has been associated with airway remodeling, induced by cigarette smoke extract have been demonstrated [34]. Budesonide can inhibit chlorine-induced airway fibrosis [35]. And the effect of LTRA on attenuate airway remodeling by inhibiting TGF-β/Smad signaling has been also demonstrated [36]. However, recent studies have also focused on the ineffective of glucocorticoid in treating airway remodeling [37, 38]. Our results showed that neither glucocorticoid or montelukast sodium can reverse the changes of EMT markers induced by TGF-β1/IL-1β. This may explain why neutrophilic asthmatics with higher airway IL-1β expression had poor lung function improvement although they also received sufficient standardized therapy.

Previous studies have demonstrated the critical role of MAPK signaling pathways (such as ERK1/2, JNK, and p38 signaling pathways) and other signaling pathways (such as PI3K/AKT, NF-κb, and Smads signaling pathways) in EMT induced by TGF-β1 or other cytokines and airway remodeling [39–46].Some of drugs, chemicals, and probiotics can alleviate airway remodeling through attenuation these signaling pathways [38, 47–50]. To further investigate the mechanism of IL-1β augmenting TGF-β1 induced EMT, candidate signaling pathways related proteins were detected. In our cellular model, we find that TGF-β1combine with IL-1β, but not TGF-β1 alone, induces ERK1/2, JNK1/2, and p38 activation. Inhibitors of MEK, p-JNK1/2, and p-p38 can reverse the changes of EMT markers induced by TGF-β1/IL-1β. These results indicate that IL-1β may act through MAPK signaling pathways to augment TGF-β1 induced EMT.

Evidences show that IL-17A is associated with airway neutrophilic inflammation in asthmatic patients through promoting neutrophils recruitment and accumulation by inducing cytokines released from structural cells, such as bronchial epithelial and venous endothelial cells [51]. In this study, the expression of IL-17A mRNA was not increased in sputum of neutrophilic asthma in this study. The reasons may be due to several issues. Firstly, the sputum may differ from the epithelial cells or bronchial biopsy in IL-17 expression. Previous literatures reported gene expressions of IL-17 response signatures in epithelial brushing or bronchial biopsy can be used to identify an IL-17–high asthma phenotype [52]. However, Manise et al. [53] have reported that IL-17 levels in sputum supernatant of neutrophilic asthmatics were similar to those of eosinophilic or pauci-granulocytic asthmatics. Another study showed that, in steroid-naïve asthmatic patients, IL-17A mRNA levels in sputum did not significantly correlate with the percentage of neutrophil in sputum [54]. In our study, all patients were steroid-naïve at the time of enrollment. Secondly, in this study, the IL-17A mRNA significantly increased in the mixed phenotype of asthma patients compared with pauci-granulocytic phenotype. Molet et al. [55] have demonstrated that eosinophils in asthmatic airway expressed IL-17. Wakashin et al. [56] showed that IL-23/IL-17A axis enhances Th2 cytokine mediated eosinophil recruitment into the airways. A novel subset of dual-positive Th2/Th17 cells co-expressing IL-4 and IL-17 was identified and the number of this subset cells was positively correlated with eosinophil count in BALF [56]. In addition, the frequency of circulation Th2 cells that produce both IL-17A and classical Th2 cytokines (IL-4, IL-5, and IL-13) is higher in patients with atopic asthma than healthy controls [57]. These studies showed a significant cross-talk exists between IL-17 and eosinophilic inflammation which may explain why IL-17A mRNA significantly increased in asthmatics with mixed-granulocytic phenotype, a special phenotype with both neutrophilic and eosinophilic inflammation. Finally, the mRNA expression of IL-17A may not be consistent with its protein expression. We have tried to measure IL-17 protein in sputum supernatant by ELISA. However, sputum IL-17 was below the limit of detection in all samples. This may be caused by DTT (0.1% concentration) used in our study decreased the level of IL-17 in sputum supernatant [58] and made it below the limit of detection [59].

Of note, in our study, healthy controls were younger than asthmatic patients, although the difference of age among the four asthmatic subgroups were not significant. While the six genes included in this study were carefully chosen to characterize the innate and type-2 adaptive immunity, testing a larger number of cytokines using high-throughput approaches will provide a full understanding of the immune network in neutrophilic asthmatics. We also note, previous study defined persistent inflammation as stability of elevated inflammatory markers over one year [60], thus sampling only once may not represent persistent inflammation levels. Finally, the subjects in this study are relatively mild asthma patients which may differ with the severe asthma patients in the mechanism of airway remodeling.

## Conclusions

In conclusion, we have identified IL-1β mRNA in induced sputum of untreated asthmatics as an important predictor for poor lung function improvement at response of 6-month standardized therapy in neutrophilic asthmatics. The underlying mechanism is related to IL-1β augmenting TGF-β1 induced EMT through MAPK signaling pathways, and this process cannot be abrogated by glucocorticoid or LTRA.

## Supplementary Information

**Additional file 1: Figure S1.** The baseline sputum cell mRNA levels among four subgroups of asthmatic patients.

**Additional file 2: Table S1.** The correlations among baseline sputum cell mRNA expression and induced sputum cell counts of asthmatic patients (n = 115).

**Additional file 3: Table S2.** Baseline demographic characteristics of asthmatic patients with spirometry follow up (n = 54).

**Additional file 4: Figure S2.** Lung function and ACT scores after 6 months standardized therapy among four subgroups of asthmatic patients.

**Additional file 5: Table S3.** The correlations among baseline sputum inflammatory factors and spirometry change in asthmatic patients.

**Additional file 6: Figure S3.** The thickness of RBM was increased in asthmatics and positively correlated with neutrophil percentage in BALF.

**Additional file 7: Figure S4.** The effect of glucocorticoid and montelukast sodium on TGF-β1 combined with IL-1β-induced EMT in A549 cells.

## Data Availability

The datasets generated during and/or analyzed during the current study are available from the corresponding author on reasonable request.

## Abbreviations

ACT: Asthma Control Test
BALF: Bronchoalveolar lavage fluid
BMI: Body mass index
ELISA: Enzyme-linked immunosorbent assay
EMT: Epithelial-mesenchymal transition
FEV_1_: Forced expiratory volume in one second
FOXP3: Forkhead box protein 3
IFN: Interferon
IL: Interleukin
LTRA: Leukotriene receptor antagonists
RBM: Reticular basement membrane
RT-qPCR: Quantitative reverse transcription
TGF: Transforming growth factor
