# Supplementary material for: IL-1β augments TGF-β inducing epithelial-mesenchymal transition of epithelial cells and associates with poor pulmonary function improvement in neutrophilic asthmatics
Source: Respir Res. 2021 Aug 3;22:216. doi: 10.1186/s12931-021-01808-7 (PMC8336269; doi:10.1186/s12931-021-01808-7)
Supplement: Supplementary file 2 — Additional file 2: Table S1. The correlations among baseline sputum cell mRNA expression and induced sputum cell counts of asthmatic patients (n = 115). [file 12931_2021_1808_MOESM2_ESM.docx]

**Table S1.** The correlations among baseline sputum cell mRNA expression and induced sputum cell counts of asthmatic patients (n = 115).

|  | Spearman | IL-1β | IL-27 | IFN-γ | IL-5 | Eos% | Neu% | Lym% | Mac% |
| --- | --- | --- | --- | --- | --- | --- | --- | --- | --- |
| IL-1β | Correlation Coefficient | 1.000 |  |  |  |  |  |  |  |
|  | Sig. (2-tailed) | - |  |  |  |  |  |  |  |
| IL-27 | Correlation Coefficient | 0.073 | 1.000 |  |  |  |  |  |  |
|  | Sig. (2-tailed) | 0.440 | - |  |  |  |  |  |  |
| IFN-γ | Correlation Coefficient | 0.030 | **.658^**^** | 1.000 |  |  |  |  |  |
|  | Sig. (2-tailed) | 0.753 | **0.000** | - |  |  |  |  |  |
| IL-5 | Correlation Coefficient | -0.126 | **.442^**^** | **.564^**^** | 1.000 |  |  |  |  |
|  | Sig. (2-tailed) | 0.183 | **0.000** | **0.000** | - |  |  |  |  |
| Eos% | Correlation Coefficient | **-.224^*^** | -0.156 | 0.053 | **.370^**^** | 1.000 |  |  |  |
|  | Sig. (2-tailed) | **0.017** | 0.098 | 0.578 | **0.000** | - |  |  |  |
| Neu% | Correlation Coefficient | **.459^**^** | 0.110 | -0.133 | -0.177 | **-.196^*^** | 1.000 |  |  |
|  | Sig. (2-tailed) | **0.000** | 0.243 | 0.158 | 0.060 | **0.035** | - |  |  |
| Lym% | Correlation Coefficient | -0.012 | -0.047 | 0.019 | 0.005 | 0.006 | **-.256^**^** | 1.000 |  |
|  | Sig. (2-tailed) | 0.897 | 0.618 | 0.837 | 0.955 | 0.947 | **0.006** | - |  |
| Mac% | Correlation Coefficient | **-.230^*^** | 0.049 | 0.031 | -0.052 | **-.270^**^** | **-.652^**^** | 0.067 | 1.000 |
|  | Sig. (2-tailed) | **0.014** | 0.601 | 0.741 | 0.581 | **0.003** | **0.000** | 0.475 | - |

Simplified version

|  | Spearman | Eos% | Neu% | Lym% | Mac% |
| --- | --- | --- | --- | --- | --- |
| IL-1β | Correlation Coefficient | **-.224*** | **.459**** | -0.012 | **-.230*** |
|  | Sig. (2-tailed) | **0.017** | **0.000** | 0.897 | **0.014** |
| IL-27 | Correlation Coefficient | -0.156 | 0.110 | -0.047 | 0.049 |
|  | Sig. (2-tailed) | 0.098 | 0.243 | 0.618 | 0.601 |
| IFN-γ | Correlation Coefficient | 0.053 | -0.133 | 0.019 | 0.031 |
|  | Sig. (2-tailed) | 0.578 | 0.158 | 0.837 | 0.741 |
| IL-5 | Correlation Coefficient | **.370**** | -0.177 | 0.005 | -0.052 |
|  | Sig. (2-tailed) | **0.000** | 0.060 | 0.955 | 0.581 |

Spearman R-values and *p*-values are indicated, values in bold are statistically significant.

*Correlation is significant at the 0.05 level (2-tailed).

**Correlation is significant at the 0.01 level (2-tailed)
