# Supplementary material for: IL-1β augments TGF-β inducing epithelial-mesenchymal transition of epithelial cells and associates with poor pulmonary function improvement in neutrophilic asthmatics
Source: Respir Res. 2021 Aug 3;22:216. doi: 10.1186/s12931-021-01808-7 (PMC8336269; doi:10.1186/s12931-021-01808-7)
Supplement: Supplementary file 3 — Additional file 3: Table S2. Baseline demographic characteristics of asthmatic patients with spirometry follow up (n = 54). [file 12931_2021_1808_MOESM3_ESM.docx]

**Table S2. Baseline demographic characteristics of asthmatic patients with spirometry follow up (n = 54)**

|  | Eosinophilic  (n = 20) | Neutrophilic  (n = 12) | Mixed  (n = 5) | Paucigranulocytic  (n = 17) | Overall *P* value |
| --- | --- | --- | --- | --- | --- |
| Age (y) | 44 (27-51) | 42 (33-50) | 49 (34-51) | 45 (31-51) | 0.9083 |
| Male sex n. (%) | 11 (55) | 3 (25) | 3 (60) | 7 (41) | 0.334 |
| BMI (kg/m^2^) | 22.7± 3.5 | 22.0± 2.3 | 23.9± 2.1 | 23.7± 4.0 | 0.485 |
| Smoker n. (%) | 3 (15) | 1 (8) | 1 (20) | 6 (35) | 0.288 |
| Atopy n. (%) | 12 (60) | 4 (33) | 2 (40) | 6 (35) | 0.365 |
| Asthma course (y) | 2.3 (1.0-5.5) | 2.0 (0.4-3.0) | 4.0 (1.5-9.5) | 0.5 (0.3-6.5) | 0.1075 |
| Blood eosinophils (%) | 6.4 (3.3-8.4) £ | 2.9 (1.3-5.1) | 6.8 (5.8-9.3) £ | 1.8 (1.2-3.4) | < 0.001 |
| Blood neutrophils (%) | 58.2 (49.4-64.7) | 59.6 (54.1-65.7) | 56.0 (52.3-65.9) | 56.2 (54.4-64.6) | 0.887 |
| FEV_1_ (L) | 2.57 ± 0.65 | 2.60 ± 0.72 | 2.84 ± 1.04 | 2.66 ± 0.71 | 0.891 |
| FEV_1_ (%) | 85.0 ± 17.6 | 90.2 ± 14.2 | 88.6 ± 24.3 | 87.2 ± 16.8 | 0.873 |
| FEV_1_/FVC (%) | 68.7 ± 7.7 | 73.1 ± 9.0 | 67.3 ± 9.0 | 70.3 ± 11.1­­ | 0.537 |
| Serum IgE (IU/ml) | 220 (136-583) £ | 61 (33-111) | 233 (28-478) | 46 (9-149) | 0.006 |
| FE_NO_ (ppb) | 95 (45-121) £ | 27 (17-45) | 72 (37-136) | 24 (11-46) | 0.002 |
| ACT score | 16.0 (13.5-17.8) £ | 17.0 (16.0-19.0) | 17.0 (15.5-20.5) | 19.0 (18.0-19.0) | 0.029 |
| Induced sputum characteristics |  |  |  |  |  |
| Macrophages (%) | 22.1 (9.7-33.2)£ | 6.5 (1.9-15.4) £ | 5.2 (2.3-12.4) £ | 47.2 (37.2-51.8) | < 0.001 |
| Neutrophils (%) | 31.7 (22.3-44.9) ¶ | 88.1 (73.1-91.8) £k | 71.3 (67.6-74.4) £ | 39.8 (32.7-45.5) | < 0.001 |
| Eosinophils (%) | 22.6 (8.8-43.1) †£ | 0.7 (0.1-1.6) | 15.1 (9.7-18.8) £ | 0.4 (0-1.1) | < 0.001 |
| Lymphocytes (%) | 7.7 (5.3-13.2) | 3.8 (2.8-7.2) | 4.6 (3.4-9.9) | 10.7 (5.7-21.0) | 0.034 |

Normal data are expressed as mean ± SD and non-normal data are described as median (IQR). Multiple groups were compared using one-way analysis of variance (ANOVA) with a Bonferroni correction (normal data) or a Kruskal-Wallis test with a Dunn intergroup comparison (non-normal data). The Levene method was used to test for multiple-sample homogeneity of variance, and Welch method was performed when data are heterogeneous. The χ2 or Fisher exact tests were used to compare ratios;

BMI, body mass index; FE_NO_, fraction of exhaled nitric oxide; ACT, Asthma Control Test; IQR, interquartile range; NA, not available.

Atopy was defined aswas defined as at least one specific IgE (≥ 0.35 kUI/L) toward common aeroallergens, a positive skin prick test response, or both.

Smoker was defined as current smokers or ex-smokers who had the smoking history of more than five pack-years or quit smoking for less than six months.

k, *p* < 0.05 versus patients with eosinophilic asthma.

†, *p* < 0.05 versus patients with neutrophilic asthma.

¶, *p* < 0.05 versus patients with mixed asthma.

£, *p* < 0.05 versus patients with Paucigranulocytic asthma.
