# Supplementary material for: IL-1β augments TGF-β inducing epithelial-mesenchymal transition of epithelial cells and associates with poor pulmonary function improvement in neutrophilic asthmatics
Source: Respir Res. 2021 Aug 3;22:216. doi: 10.1186/s12931-021-01808-7 (PMC8336269; doi:10.1186/s12931-021-01808-7)
Supplement: Supplementary file 4 — Additional file 4: Figure S2. Lung function and ACT scores after 6 months standardized therapy among four subgroups of asthmatic patients. [file 12931_2021_1808_MOESM4_ESM.pptx]

## Slide 1
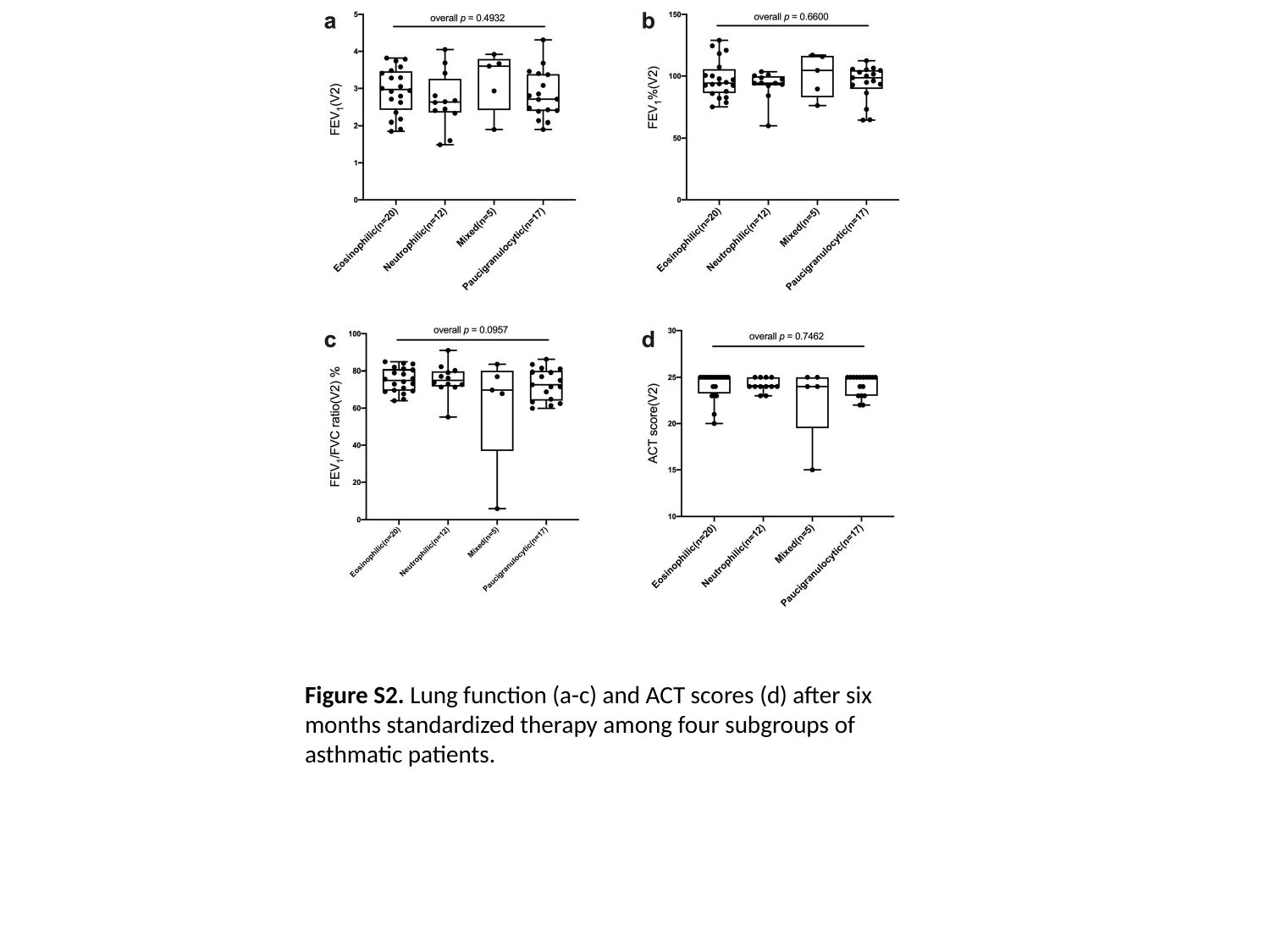

Figure S2. Lung function (a-c) and ACT scores (d) after six months standardized therapy among four subgroups of asthmatic patients.
