# Supplementary material for: IL-1β augments TGF-β inducing epithelial-mesenchymal transition of epithelial cells and associates with poor pulmonary function improvement in neutrophilic asthmatics
Source: Respir Res. 2021 Aug 3;22:216. doi: 10.1186/s12931-021-01808-7 (PMC8336269; doi:10.1186/s12931-021-01808-7)
Supplement: Supplementary file 5 — Additional file 5: Table S3. The correlations among baseline sputum inflammatory factors and spirometry change in asthmatic patients. [file 12931_2021_1808_MOESM5_ESM.docx]

**Table S3.** The correlations among baseline sputum inflammatory factors and spirometry change in asthmatic patients, Simplified version.

**3.1** all asthmatic patients with delta spirometry (n = 54)

|  | Spearman | ΔFEV_1(V2-V1)_ | ΔFEV_1_%_(V2-V1)_ | ΔFEV_1_/FEV_1(V1)_ |
| --- | --- | --- | --- | --- |
| IL-1β protein | Correlation Coefficient | -0.224 | -0.271* | -0.197 |
|  | Sig. (2-tailed) | 0.107 | 0.049 | 0.156 |
| IL-1β mRNA | Correlation Coefficient | -0.316* | -0.339* | -0.282* |
|  | Sig. (2-tailed) | 0.020 | 0.012 | 0.039 |
| IL-27 mRNA | Correlation Coefficient | -0.042 | -0.010 | -0.018 |
|  | Sig. (2-tailed) | 0.765 | 0.944 | 0.898 |
| IFN-γ mRNA | Correlation Coefficient | -0.019 | -0.009 | 0.044 |
|  | Sig. (2-tailed) | 0.890 | 0.949 | 0.753 |
| IL-5 mRNA | Correlation Coefficient | 0.052 | 0.072 | 0.077 |
|  | Sig. (2-tailed) | 0.710 | 0.606 | 0.579 |
| Eos% | Correlation Coefficient | 0.383** | 0.382** | 0.387** |
|  | Sig. (2-tailed) | 0.004 | 0.004 | 0.004 |
| Neu% | Correlation Coefficient | -0.265 | -0.266 | -0.233 |
|  | Sig. (2-tailed) | 0.053 | 0.052 | 0.090 |

**3.2** Eosinophilic asthma group (n = 20)

|  | Spearman | ΔFEV_1(V2-V1)_ | ΔFEV_1_%_(V2-V1)_ | ΔFEV_1_/FEV_1(V1_) |
| --- | --- | --- | --- | --- |
| IL-1β protein | Correlation Coefficient | 0.138 | 0.100 | 0.204 |
|  | Sig. (2-tailed) | 0.573 | 0.683 | 0.403 |
| IL-1β mRNA | Correlation Coefficient | -0.114 | 0.045 | 0.005 |
|  | Sig. (2-tailed) | 0.633 | 0.850 | 0.985 |
| IL-27 mRNA | Correlation Coefficient | 0.102 | 0.251 | 0.104 |
|  | Sig. (2-tailed) | 0.668 | 0.286 | 0.663 |
| IFN-γ mRNA | Correlation Coefficient | 0.121 | 0.177 | 0.092 |
|  | Sig. (2-tailed) | 0.611 | 0.454 | 0.701 |
| IL-5 mRNA | Correlation Coefficient | 0.061 | -0.011 | -0.023 |
|  | Sig. (2-tailed) | 0.799 | 0.965 | 0.925 |
| Eos% | Correlation Coefficient | 0.299 | 0.272 | 0.198 |
|  | Sig. (2-tailed) | 0.200 | 0.246 | 0.402 |
| Neu% | Correlation Coefficient | -0.371 | -0.135 | -0.221 |
|  | Sig. (2-tailed) | 0.107 | 0.569 | 0.349 |

**3.3** Neutrophilic asthma group (n = 12)

|  | Spearman | ΔFEV_1(V2-V1)_ | ΔFEV_1_%(V2-V1) | ΔFEV_1_/FEV_1(V1)_ |
| --- | --- | --- | --- | --- |
| IL-1β protein | Correlation Coefficient | -0.147 | -0.140 | -0.014 |
|  | Sig. (2-tailed) | 0.646 | 0.667 | 0.974 |
| IL-1β mRNA | Correlation Coefficient | -0.623* | -0.692* | ­-0.566 |
|  | Sig. (2-tailed) | 0.034 | 0.016 | 0.060 |
| IL-27 mRNA | Correlation Coefficient | 0.053 | 0.133 | 0.021 |
|  | Sig. (2-tailed) | 0.873 | 0.683 | 0.956 |
| IFN-γ mRNA | Correlation Coefficient | -0.361 | -0.280 | -0.287 |
|  | Sig. (2-tailed) | 0.248 | 0.379 | 0.366 |
| IL-5 mRNA | Correlation Coefficient | -0.242 | -0.224 | -0.028 |
|  | Sig. (2-tailed) | 0.446 | 0.485 | 0.939 |
| Eos% | Correlation Coefficient | -0.112 | -0.130 | 0.049 |
|  | Sig. (2-tailed) | 0.726 | 0.687 | 0.881 |
| Neu% | Correlation Coefficient | 0.179 | 0.119 | 0.315 |
|  | Sig. (2-tailed) | 0.576 | 0.716 | 0.319 |

**3.4** Paucigranulocytic asthma group (n = 17)

|  | Spearman | ΔFEV_1(V2-V1)_ | ΔFEV_1_%_(V2-V1)_ | ΔFEV_1_/FEV_1(V1)_ |
| --- | --- | --- | --- | --- |
| IL-1β protein | Correlation Coefficient | -0.387 | -0.495* | -0.395 |
|  | Sig. (2-tailed) | 0.125 | 0.045 | 0.118 |
| IL-1β mRNA | Correlation Coefficient | -0.140 | -0.287 | -0.174 |
|  | Sig. (2-tailed) | 0.590 | 0.264 | 0.503 |
| IL-27 mRNA | Correlation Coefficient | -0.060 | -0.113 | 0.012 |
|  | Sig. (2-tailed) | 0.818 | 0.667 | 0.966 |
| IFN-γ mRNA | Correlation Coefficient | -0.047 | -0.179 | 0.000 |
|  | Sig. (2-tailed) | 0.859 | 0.491 | 1.000 |
| IL-5 mRNA | Correlation Coefficient | -0.230 | -0.081 | -0.211 |
|  | Sig. (2-tailed) | 0.373 | 0.758 | 0.415 |
| Eos% | Correlation Coefficient | 0.288 | 0.074 | 0.282 |
|  | Sig. (2-tailed) | 0.259 | 0.777 | 0.270 |
| Neu% | Correlation Coefficient | -0.075 | -0.230 | -0.061 |
|  | Sig. (2-tailed) | 0.774 | 0.372 | 0.817 |

Spearman R-values and *p*-values are indicated, values in bold are statistically significant.

*Correlation is significant at the 0.05 level (2-tailed).

**Correlation is significant at the 0.01 level (2-tailed)
