# Supplementary material for: IL-1β augments TGF-β inducing epithelial-mesenchymal transition of epithelial cells and associates with poor pulmonary function improvement in neutrophilic asthmatics
Source: Respir Res. 2021 Aug 3;22:216. doi: 10.1186/s12931-021-01808-7 (PMC8336269; doi:10.1186/s12931-021-01808-7)
Supplement: Supplementary file 6 — Additional file 6: Figure S3. The thickness of RBM was increased in asthmatics and positively correlated with neutrophil percentage in BALF. [file 12931_2021_1808_MOESM6_ESM.pptx]

## Slide 1
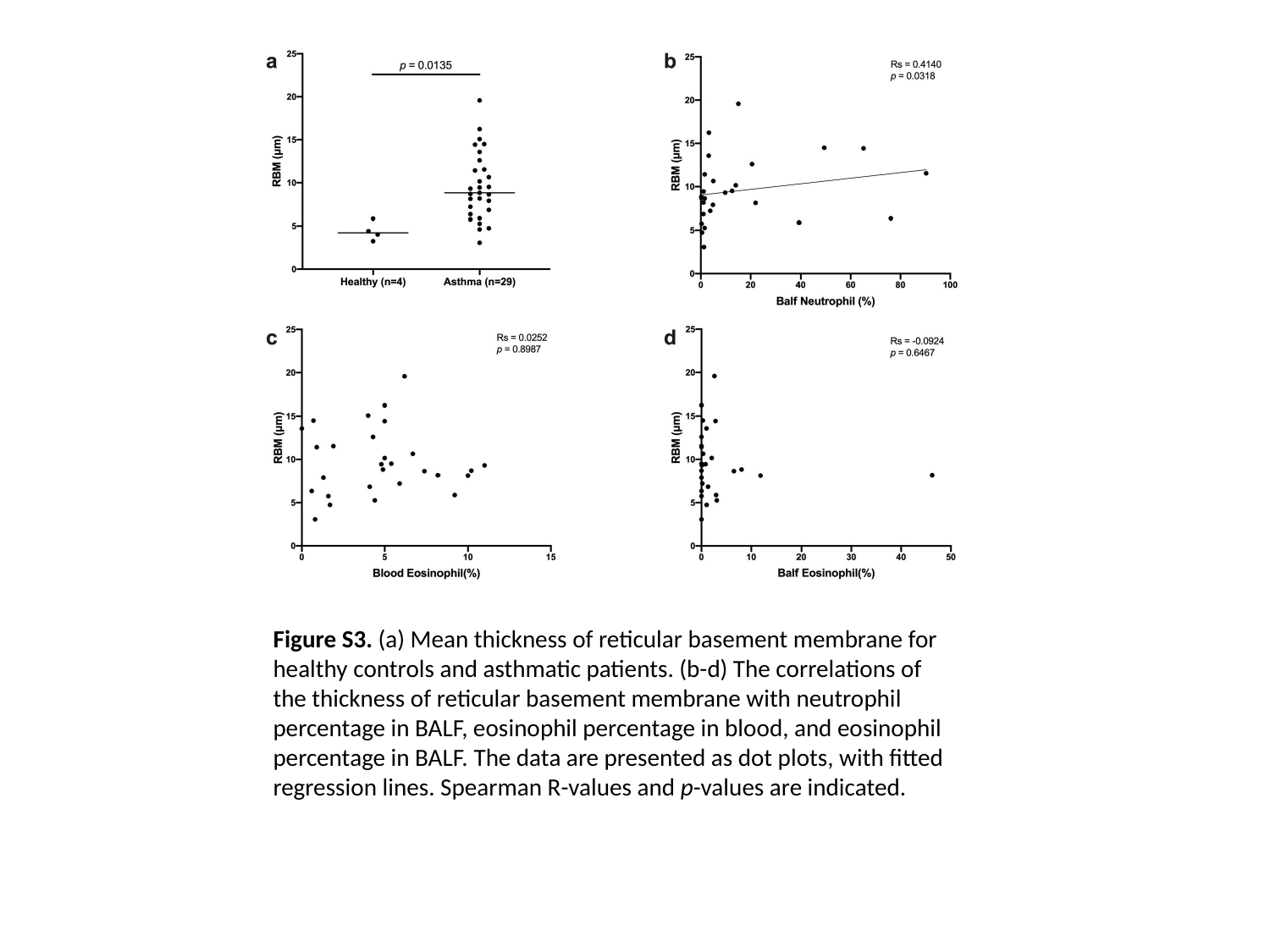

Figure S3. (a) Mean thickness of reticular basement membrane for healthy controls and asthmatic patients. (b-d) The correlations of the thickness of reticular basement membrane with neutrophil percentage in BALF, eosinophil percentage in blood, and eosinophil percentage in BALF. The data are presented as dot plots, with fitted regression lines. Spearman R-values and p-values are indicated.
