# Supplementary material for: IL-1β augments TGF-β inducing epithelial-mesenchymal transition of epithelial cells and associates with poor pulmonary function improvement in neutrophilic asthmatics
Source: Respir Res. 2021 Aug 3;22:216. doi: 10.1186/s12931-021-01808-7 (PMC8336269; doi:10.1186/s12931-021-01808-7)
Supplement: Supplementary file 7 — Additional file 7: Figure S4. The effect of glucocorticoid and montelukast sodium on TGF-β1 combined with IL-1β-induced EMT in A549 cells. [file 12931_2021_1808_MOESM7_ESM.pptx]

## Slide 1
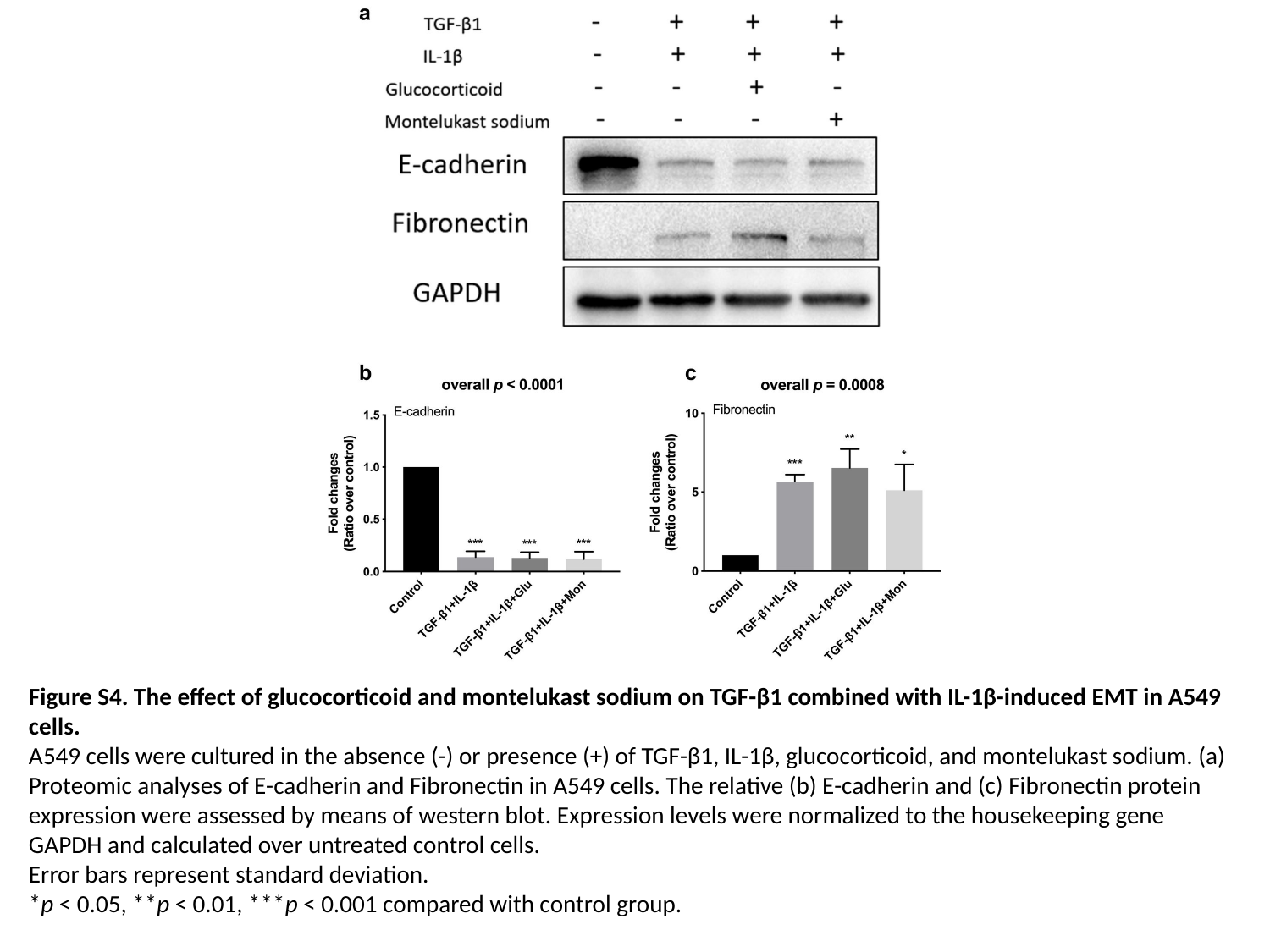

Figure S4. The effect of glucocorticoid and montelukast sodium on TGF-β1 combined with IL-1β-induced EMT in A549 cells.
A549 cells were cultured in the absence (-) or presence (+) of TGF-β1, IL-1β, glucocorticoid, and montelukast sodium. (a) Proteomic analyses of E-cadherin and Fibronectin in A549 cells. The relative (b) E-cadherin and (c) Fibronectin protein expression were assessed by means of western blot. Expression levels were normalized to the housekeeping gene GAPDH and calculated over untreated control cells.
Error bars represent standard deviation.
*p < 0.05, **p < 0.01, ***p < 0.001 compared with control group.
